# Reactivation of Notch signaling is required for cardiac valve regeneration

**DOI:** 10.1038/s41598-019-52558-y

**Published:** 2019-11-05

**Authors:** Panagiotis Kefalos, Adamantia Agalou, Koichi Kawakami, Dimitris Beis

**Affiliations:** 1 Zebrafish Disease Model lab, Center for Experimental Surgery, Clinical and Translational Research, Biomedical Research Foundation, Academy of Athens, Athens, GR11527 Greece; 2 Department of Biology, University of Patras, Patras, GR26504 Greece; 3 Division of Molecular and Developmental Biology, National Institute of Genetics, and Department of Genetics, SOKENDAI (The Graduate University for Advanced Studies), Mishima, 411-8540 Japan

**Keywords:** Disease model, Zebrafish, Regeneration

## Abstract

Cardiac Valve Disease is one of the most common heart disorders with an emerging epidemic of cardiac valve degeneration due to aging. Zebrafish can regenerate most of their organs, including their heart. We aimed to explore the regenerative potential of cardiac valves and the underlying molecular mechanisms involved. We used an inducible, tissue-specific system of chemogenetic ablation and showed that zebrafish can also regenerate their cardiac valves. Upon valvular damage at larval stages, the intracardiac flow pattern becomes reminiscent of the early embryonic stages, exhibiting an increase in the retrograde flow fraction through the atrioventricular canal. As a result of the altered hemodynamics, *notch1b* and *klf2a* expression are ectopically upregulated, adopting the expression pattern of earlier developmental stages. We find that Notch signaling is re-activated upon valvular damage both at larval and adult stages and that it is required during the initial regeneration phase of cardiac valves. Our results introduce an animal model of cardiac valve specific ablation and regeneration.

## Introduction

Zebrafish valve development is a unique system where we can study the interactions between morphogenesis and function of the heart. Cardiac valves develop after the heart starts beating and since zebrafish hearts have a single atrium and ventricle the cells are easy to trace and follow at single-cell resolution^1^. Zebrafish embryos can survive even in the absence of a fully functional cardiovascular system for several days because they receive enough oxygen by passive diffusion from the water they grow. This feature enables the study of severe heart mutations that would be instantly lethal in mammalian embryos and provide the ability to manipulate cardiac function and intracardiac flow dynamics^2^. Taking advantage of surgical and pharmacological manipulations as well as of mutants with defective myocardial contractility (*silent heart*, *weak atrium*) and altered intracardiac flow dynamic due to changes in heart geometry (*southpaw*), zebrafish has been pivotal in studying these interactions *in vivo*^3–8^. These studies are largely facilitated by the ability to do high-resolution imaging at the cellular level, using high-speed cameras and/or Selective Plane Illumination Microscopy (SPIM) imaging and image reconstruction^8,9^, allowing a better understanding of the effects of shear-stress on cardiac valve cells.

Notch is one of the most ancient and conserved signaling pathways^10^ and has been connected to cardiac and, more specifically, valve development in several occasions. Mutations in NOTCH signaling elements cause congenital heart defects and NOTCH1 heterozygous Humans show increased risk of bicuspid aortic valve and aortic valve calcification^11^. Notch remains active in the valve region throughout the lifetime of an organism (including zebrafish) in the high shear-stress regions and acts to suppress signaling pathways that could lead to valve calcification^12,13^. Aging is the highest risk factor for valve calcification and in fact mice with shortened telomeres exhibit the age-dependent human phenotypes from neonatal stages^14^. Zebrafish emerged as a valuable model to study organ regeneration. Several tissues such as the fin^15^, the myocardium^16^, the pancreas^17,18^ and most other organs tested^19^ showed regenerative capability and the signaling pathways and mechanisms identified are now being tested in mammalian systems.

Tissue-engineered heart valves with regenerative capacities are expected to be a promising alternative to the current surgery treatments, particularly for young patients^20^. We set out to explore the regenerative capacity of cardiac valves and identify signaling pathways that are necessary for this. Since zebrafish cardiac valve cells even in adult stages are not available to surgery, and there are no available valve specific promoters, we developed an inducible two component system using specific GAL4 driver lines to express the Nitroreductase gene in valve cells and induce their damage by adding Metronidazole in the fish water. We found that reactivation of the Notch signaling pathway, following valvular damage is necessary for the initiation of valve regeneration in both larval and adult stages.

## Results

### An inducible valve ablation system reveals the regenerative potential of zebrafish cardiac valves

**Figure 1:**
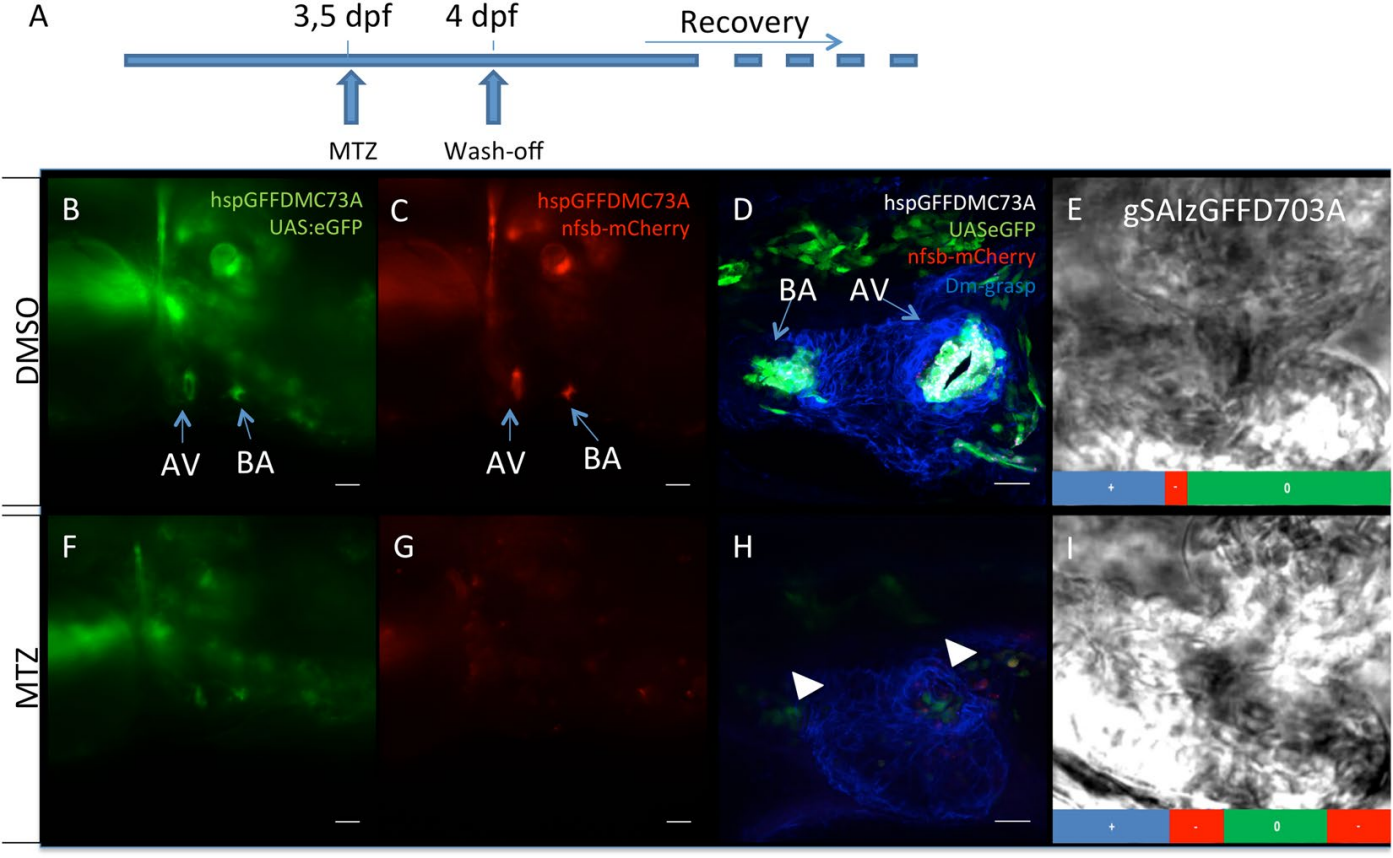
Chemically induced genetic cell ablation of zebrafish larval cardiac valves. (**A**) Outline of chemical treatment of UAS*-E1b:NfsB-*mCherry transgenic embryos with Metronidazole (MTZ). Metronidazole was applied at 3.5 dpf embryos and washed-off at 4 dpf. Embryos were then left for recovery in order to observe regeneration process. (**B,C**) Gfp and mCherry Gal4 driven expression in 4 dpf cardiac valves (Arrows). AV: atrioventricular canal, (BA): bulbus arteriosus. Scale bars: 100 μm. (**D**) Confocal z stack projection of a double transgenic Tg(*hspGFFDMC73A/UAS-E1b:NfsB-mCherry*) embryonic heart stained with cell-cell adhesion marker Dm:grasp (blue). Scale bar: 20 μm (**F,G**) Gfp and mCherry Gal4 driven expression in 4 dpf cardiac valves of MTZ treated embryos. Injury is observed at AV canal and BA nfsb-mCherry+ cells that have been ablated. Scale bars: 100 μm. (**H**) Confocal z-stack projection of a double transgenic Tg(*hspGFFDMC73A/UAS-E1b:NfsB-mCherry*) heart treated with MTZ and stained with cell-cell adhesion marker Dm-grasp (blue). Arrowheads depict the position where AV canal and BA differentiated cells should be. Scale bar: 20 μm. (**E–I**) Snapshots of high frame live videos of a DMSO treated and a MTZ-treated Tg(*gSAIzGFFD703A/UAS-E1b:NfsB-mCherry*) embryo 4 dpf, respectively (S. Movies 3 and 4). Bars show the haemodynamic flow patterns at the atriovantricular canal of uninjured and injured valves per heartbeat. The number of frames per total frames of a heartbeat was measured for forward flow (+) no flow (0) and reverse flow (−). The reverse flow fraction is increased 4,91 times (quantified from n = 8 ctrl embryos and 8 MTZ-treated embryos. p < 0.001 using paired t-test) upon valve ablation. +: forward fraction. 0: null fraction. −: reverse fraction.

**Figure 2:**
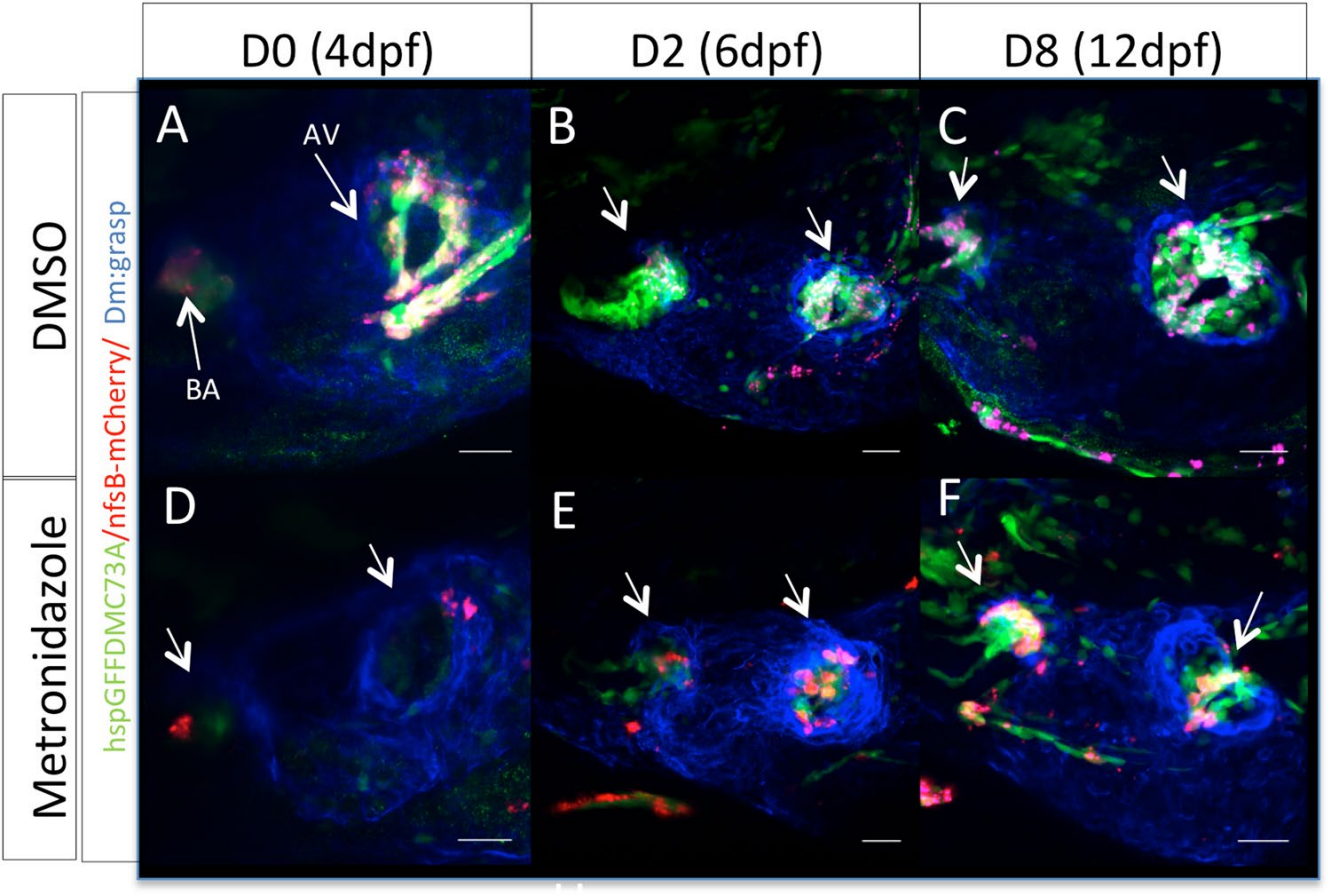
Zebrafish embryonic valves can regenerate. (**A–C**) Confocal z-stacks of untreated Tg(*hspGFFDMC73A/UAS-E1b:NfsB-mCherry*) larvae at 4 dpf, 6 dpf and 12 dpf respectively. (**D–F**) Confocal z-stacks of MTZ treated Tg(*hspGFFDMC73A/UAS-E1b:NfsB-mCherry*) and larvae at 4 dpf, 6 dpf and 12 dpf respectively. Arrows show the ablation of mCherry+ cells at the AV canal and OFT at 4 dpf. From 6 dpf more mCherry+ cells could be observed at the AV canal. By 12 dpf the regeneration process could be imaged, as mCherry+, AV differentiated cells were present at both valves. Scale bars: 20 microns. Each experiment was carried out at least 3 independent times with n = 10 embryos per experimental group.

There is currently a lack of well-characterized promoters for valve specific expression pattern. We have conducted a large-scale screen for the GAL4 transgenic fish using the gene trap and enhancer trap methods at the National Institute of Genetics, Mishima, Japan. In the course of the screen, we identified two cardiac valve driver lines with different pattern and intensity of expression within the cardiac valves Tg(*hspGFFDMC73A) and* Tg(*gSAIzGFFD703A)*. Tg(*hspGFFDMC73A*) drives, at 72hpf, robust expression in both Valve Endocardial Cells (VECs) (cuboidal cells at the AV boundary, arrowheads in Supplementary Fig. 1B) and future Valve Interstitial Cells (VICs) which at these stages largely overlap with the TCF positive, mesenchymal looking cells (arrows in Supplementary Fig. 1C) and this expression pattern remains on up to adulthood. Tg(*gSAIzGFFD703A*) shows weaker expression and is restricted to VECs at 72hpf (Supplementary Fig. 1D–F). We crossed both driver lines to the Tg(*UAS-E1b:NfsB-mCherry)* to test the regenerative potential of cardiac valves initially at larval stages. We optimized a metronidazole-induced ablation of valve cells at 96 hours post fertilization (hpf) (Fig. 1A), which resulted in the reproducible ablation of >80% NTR positive cells (Fig. 1 and Supplementary Fig. 2A). In order to test if cardiac valve cell ablation actually had the predicted effect on cardiac function, we quantified the intracardiac blood flow dynamics and confirmed an increase (4,91 folds) in the retrograde flow fraction of the hemodynamic pattern (Supplementary video 1 untreated, 2 ablated quantified in Fig. 1E,I and Supplementary Fig. 3A). The flow pattern of the embryos with valvular ablation is reminiscent of earlier stages (72 hpf) during valve development when the valve cells are few and they are not capable of fully preventing retrograde blood flow^6^. Chemogenetic ablation of valve cells in the Tg(*hspGFFDMC73A)* showed a higher increase of the retrograde flow fraction in accordance with the number of *mCherry* positive cells that were ablated (Supplementary Fig. 1C, compare with Supplementary Fig. 1F). The ablation was confirmed to be mediated via apoptosis, as detected in the MTZ treated embryos with TUNEL assay (Supplementary Fig. 4).We washed off metronidazole and followed larvae for the following eight days. We identified that GFP and mCherry positive cells started reappearing already at 2 days post ablation (Fig. 2A,B compare with 2D,E) and they were comparable to the untreated larvae by eight days post ablation (Fig. 2C compare with 2F and quantified in Supplementary Fig. 2B). Concerning the flow pattern at this developmental stage, no differences were observed between retrograde blood flows for both untreated (Supplementary video 3) and recovering from treatment embryos (Supplementary video 4 and quantified in Supplementary Fig. 3D).

### Notch1 is reactivated ectopically upon valvular damage and is necessary for valve regeneration

**Figure 3:**
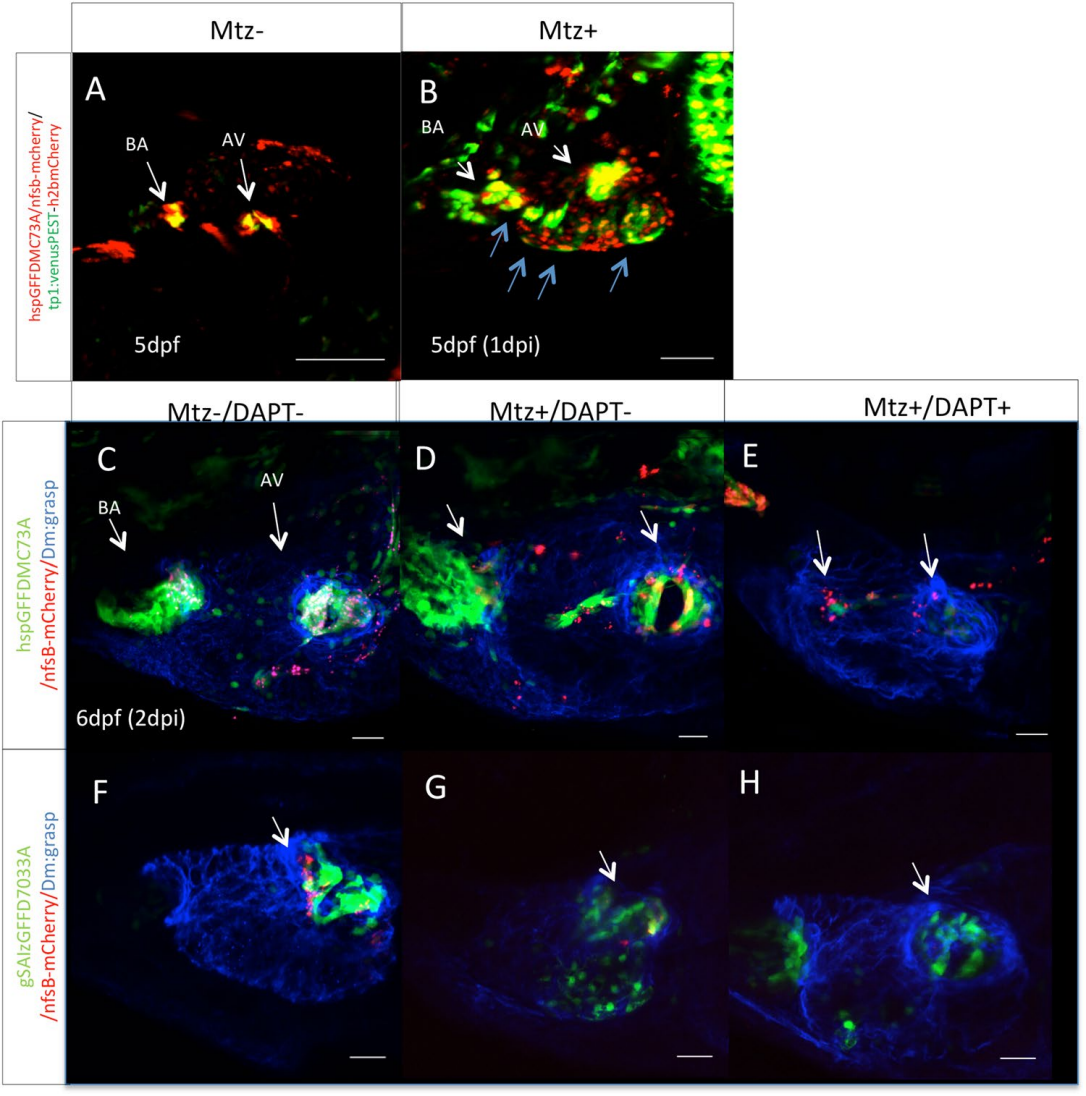
Notch signaling is activated during valve regeneration. **(A)** Confocal z-stack of a Tg(TP1:VenusPEST)/hspGFFDMC73A*: UAS-E1b:NfsB-mCherry* double transgenic embryo at 5 dpf. Tg(TP1:VenusPEST) signal of activated Notch signaling is restricted to the valves. Scale bar: 100 microns. (**B)** 1 day post MTZ treatments. Expansion of activation of Notch signaling throughout the ventricle is observed (blue arrows) Scale bar: 50microns. Confocal z-stacks of untreated Tg(*hspGFFDMC73A: UAS-E1b:NfsB-mCherry*) (**C)** and Tg(*GSAIzGFFD703A: UAS-E1b:NfsB-mCherry*) (**F)** double transgenic embryos at 6 dpf. Confocal z-stack of MTZ treated Tg(*hspGFFDMC73A: UAS-E1b:NfsB-mCherry*) (**D**), and Tg(*GSAIzGFFD703A: UAS-E1b:NfsB-mCherry*) (**G**), at 6 dpf. Confocal z-stack of MTZ treated and then during regeneration 48 hours DAPT treated Tg(*hspGFFDMC73A: UAS-E1b:NfsB-mCherry*) (**E**), and Tg(*GSAIzGFFD703A: UAS-E1b:NfsB-mCherry*) (**H**), at 6 dpf. (**I**) Each experiment was carried out at least 3 independent times with n = 10 embryos per experimental group. Scale bars: 20 micron.

In order to understand the underlying mechanisms of cardiac valve regeneration, we used several transgenic lines and *in situ* hybridization experiments. We identified that the Notch reporter line showed ectopic upregulation of expression following valvular damage. Both the shear-stress sensitive *kruppel-like factor 2a* (*klf2a)* and *notch1b* are ectopically upregulated 24 hours following valvular ablation (Supplementary Fig. 5,B, D compare with 5,A,C). In the destabilized version of the notch reporter line Tg(*TP1:VenusPEST)*, Notch signaling was activated in endocardial cells adjacent to the damaged area (Fig. 3B), while in the Tg(*TP1:h2bmCherry*) the expression domain of *mCherry* positive cells is expanded throughout the endocardium (Fig. 3B compare with 3A), since it represents the accumulating activation of ectopic Notch signaling over time. When DAPT was added in the water during the regeneration phase, no ectopic Notch activation was observed as expected. In addition, the regeneration process also halted, as monitored by the lack of reappearing *UAS-E1b:NfsB-mCherry* positive cells at the valve region (Fig. 3E compare with 3D, 3C and 3H, compare with 3G, 3F and quantified in Supplementary Fig. 6).

### Notch signaling is upregulated following adult cardiac valve ablation and is necessary for their regeneration

**Figure 4:**
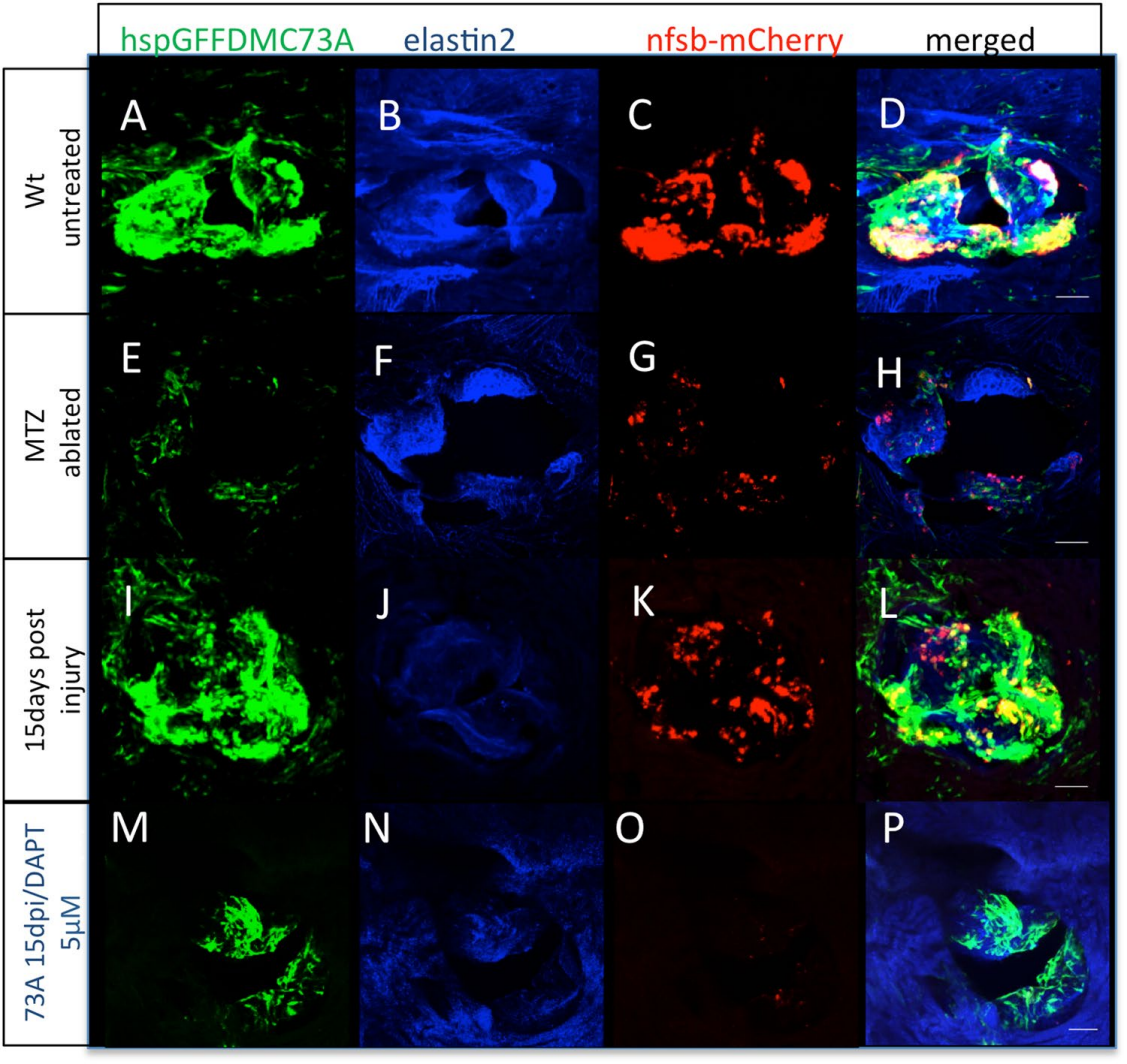
Zebrafish adult valves retain the ability to regenerate through Notch signaling reactivation. (**A–D)** Z-stack projection of a wt untreated adult valve of an hspGFFDMC73A/*UAS-E1b:NfsB-*mCherry transgenic fish stained with elastin2. (**E–H)** Valvular damage of an adult heart of a hspGFFDMC73A/*UAS-E1b:NfsB-*mCherry transgenic immediately after completion of the MTZ treatment (n = 10). (**I–L)** Regenerating valve 15 days post chemogenetic valve cell ablation (n = 13). (**M–P)** Inhibition of regeneration process in the heart of an adult heart of a hspGFFDMC73A/*UAS-E1b:NfsB-*mCherry transgenic after MTZ treatment and 15 day treatment with DAPT (n = 12). Scale bars: 50 micron.

The Tg(*hspGFFDMC73A)* driver line remains active also at adult stages in both VECs and VICs (Fig. 4A–D, Supplementary Movie 5). We added metronidazole for 12 hours and dissected hearts following the treatment to show that most of the *UAS-E1b:NfsB-mCherry* positive cells were ablated (Figure E-H, Supplementary Movie 6). Again, at this developmental stage, apoptosis was detected in MTZ treated adult valves with the TUNEL assay (Supplementary Fig. 7). We also dissected Tg(*hspGFFDMC73A/UAS-E1b:NfsB-mCherry)* hearts that carry the Tg(*TP1:VenusPEST)* transgene and showed that Notch is upregulated at the valve region (Supplementary Movies 7,8 and Supplementary Fig. 8,C,D compare with S8A,B, quantified in S8E). We allowed the fish to recover in system water or system water with 5 μM DAPT. Adult animals that were allowed to recover showed reappearance of *UAS-E1b:NfsB-mCherry* positive cells within 15 days following injury (Fig. 4I–L, Supplementary Movie 9) while we observed that Notch signaling inhibition significantly hampered the regenerative potential of adult cardiac valves (Fig. 4M–P, Supplementary Movie 10).

## Discussion

In this study, we describe the ability of cardiac valves to regenerate after chemogenetic ablation in larval and adult zebrafish. Moreover, we introduce 2 new transgenic zebrafish lines with valve expression promoters. These lines could further contribute to valve specific expression or silencing of genes of interest. Recent advances on imaging and the identification of novel cardiac valve mutants and gene networks, have helped deciphering the effect of intracardiac blood flow dynamics and shear stress on the endothelial cells that are destined to become valvular. *Klf2a* is the best-characterized flow sensitive transcription factor to date^6,21,22^. Some of its key downstream targets include the Cerebral Cavernous Malformations proteins (CCM)^23,24^ and Notch that are very important for proper valve morphogenesis^1,11,12,25–27^. Endocardial notch1b activation requires functional primary cilia^28^ and *klf2a*^6,21,22^. Klf2a has been recently shown to be also required for myocardial trabeculation integrity during development via the modulation of Fgf signaling^29^ as well as for myocardial reprogramming via its well-established endocardial hemodynamic response and Notch mediated function^30^. The CCM proteins appear to function antagonistically to the activation of β1 integrin by shear stress^31^. Knockdown of β1 integrin suppresses the cardiovascular defects of *ccm* mutant embryos^23^. In addition to the intracardiac flow dynamics and endocardial/myocardial interactions at the valve-forming region, it is worth noticing that there is extensive extracellular matrix components (ECM) also known as cardiac jelly. One of its major components is hyaluronic acid, produced by the Has2 enzyme that is tightly regulated during AV development to restrict the AV region via the BMP signaling pathway^32^ as well as via mir23^33^. Wnt signaling is also required for valve development^34,35^. Fibronectin synthesis has been shown to be flow dependent and downstream of Klf2a, coupling the mechanosensory system to ECM composition^36^.

It is becoming clear that valve regeneration would require several steps, including the proliferation of endocardial cells, their transformation to interstitial cells and the tightly regulated production of several ECM components. Therefore, cardiac valve tissue engineering (CVTE) would require the combination of optimized biomaterials with different cell types and conditioning protocols making it a very challenging process. Despite the challenges, CVTE is expected to be a promising therapeutic alternative to mechanical and bioprosthetic valves. Both of these types require life-long anticoagulative therapies and accumulate damages due to the stressful hemodynamic microenvironment of cardiac valves, since they do not contain any living cells with adaptive or regenerative potential. Such potential is particularly vital in pediatric patients whose cardiac valves need to adapt to the growing size of their hearts. The optimization of recellularization protocols to accommodate the need for replacement valves that could grow/adapt with somatic outgrowth requires the best understanding of valve development and regeneration mechanisms^20,37^. Here we addressed the initial step of valve regeneration that results from the functional consequence of a dysfunctional valve: the increase of retrograde blood flow.

**Figure 5:**
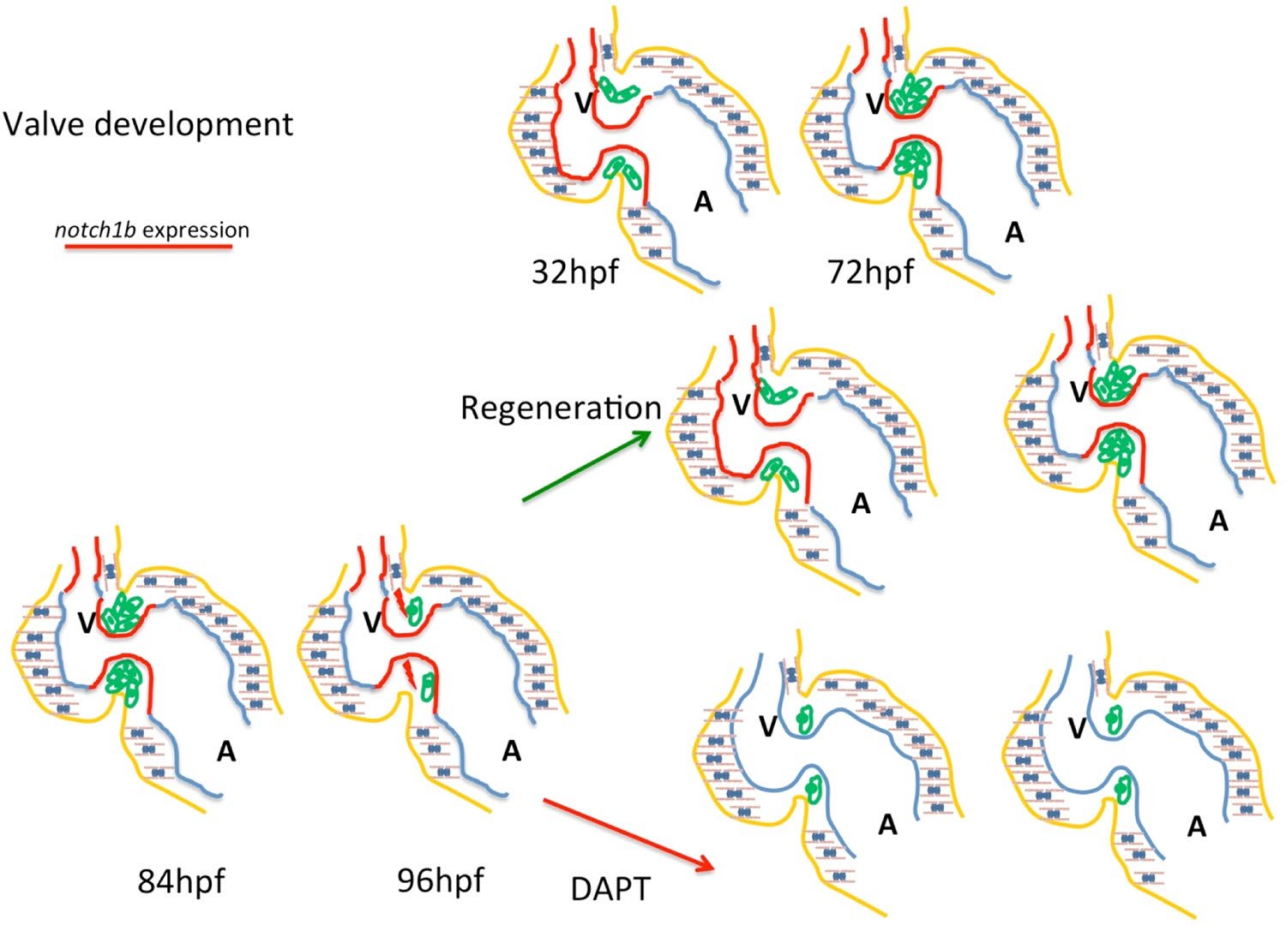
Sensing of immature intracardiac flow patterns activates a Notch mediated regenerative program, recapitulating cardiac valve development. At embryonic developmental stages a feedback loop where intracardiac flow dynamics regulate *klf2a* and *notch1b* results in the restricted expression of *notch1b* at the high sheer-stress areas of the heart: the atrioventricular valve and the outflow tract. The intracardiac flow pattern following valvular damage, is disturbed causing an increase in the retrograde blood flow fraction, reminiscent of the flow pattern of earlier stage of embryogenesis. As a result, *notch1b* is re-activated ectopically the ventricular endocardium. When cardiac valves regenerate and flow dynamics are restored, *notch1b* expression gets restricted at the atrioventricular canal and the outflow tract.

We propose a system where the immature flow patterns are sensed by the endocardial cells, and the increase of the retrograde intracardiac flow pattern, due to a dysfunctional valve, could be the stimulus for its regeneration. Following injury, the mechanosensitive transcription factor *klf2a* is upregulated, and a Notch dependent developmental program is activated for valve regeneration. This is reminiscent of the embryonic expression pattern of these signaling pathways (Fig. 5). Recapitulating development is a recurrent scenario during the regeneration process of different organs. Studies of *in vitro* cell systems that incorporate biomechanical stress and conditioning as well as *in silico* computational modeling of hemodynamics for tissue-engineered heart valve shape optimization^37^ would greatly benefit from *in vivo* experimental systems, such as the zebrafish, that show endogenous regenerative potential of their cardiac valves.

## Methods

### Zebrafish transgenic lines

gSAIzGFFD703A and hspGFFDMC73A zebrafish lines were generated during a large-scale screen for zebrafish transgenic Gal4 lines by the gene trap and enhancer trap methods at the Kawakami lab^38,39^. More information on the insertion site and expression pattern can be found at http://kawakami.lab.nig.ac.jp/ztrap/. The transgenic lines *Tg(UAS:eGFP1A)*^38^ and *Tg(UAS-E1b:NfsB-mCherry)*^*c264*^^18^, which we will refer to as NfsB-mCherry for short in figures, were crossed with the above lines to create double or triple transgenic lines. hspGFFDMC73A/*Tg(UAS-E1b:NfsB-mCherry)*^*c264*^ double transgenics were crossed with *Tg(Tp1:venus-PEST)* and/or *Tg(Tp1:h2B:mCherry)*^40^ in order to create appropriate Green/Red combinations of the transgenics to distinguish Notch signaling reporter expression following valve cell ablation. *Tg(7xTCF-Xla.Siam:nlsmCherry)*^*ia5*^ line^35^ was also used to image expression pattern and the overlap of mesenchymal-like TCF positive cells with gSAIzGFFD703A line.

### Metronidazole (Mtz) treatment of zebrafish embryos and adults

Mtz (Sigma) was diluted in embryo water/0,2%DMSO in a final concentration of 4 mM for hspGFFDMC73A/UAS*-E1b:NfsB-*mCherry 3 dpf embryos and at 10 mM for gSAIzGFFD703A/UAS*-E1b:NfsB-*mCherry 3 dpf embryos. 0,2% DMSO in embryo water was used as a control. Mtz treatment (concentration and exposure time) was optimized for the two lines and the different developmental stages to ensure reproducibility between larvae and adults as well as >80% of successful elimination of NTR+ cells. Embryos were treated for 12 and 20 hours for the 73 A and 703 A lines, respectively. Embryos were then washed-off Mtz and left for recovery for 8days. For adult transgenic fish hspGFFDMC73A/UAS*-E1b:NfsB-*mCherry Mtz treatments, fish were treated with 5 mM in system water for 12 hours and left for the mentioned interval to recover before proceeding to heart extractions. Euthanasia was carried out by prolonged immersion in water with an overdose of tricaine methane sulfonate (MS222, 300 mg/l).

### DAPT treatments

4 dpf fish were treated in 70 μΜ DAPT diluted in EW/1%DMSO until 6 dpf, where they were fixed and immunostained. Adult DAPT treatments took place as previously described with 5 μΜ DAPT final concentration in system water.

### Immunofluorescence

Zebrafish embryos were fixed in 4% PFA and stained using the Zn5 (Dm:grasp, Alcama) Ab with Alexa anti-mouse 633 as a secondary antibody. Embryos were then embedded in 4% agarose and sectioned in a vibratome at 180 μΜ sections. Adult hearts were extracted, fixed in 4%PFA, embedded, sectioned and then stained on 180 μΜ vibratome sections. Tropoelastin Ab was previously described^41^ with Alexa anti-rabbit 633 used as the secondary Ab.

### Zebrafish embryos’ live imaging and retrograde flow fraction quantification

5 dpf embryos were anesthetized in tricaine, embedded in 1% low melting agarose and imaged under a Leica confocal microscope. For brightfield videos of 4 dpf embryos ORCA Hamamatsu camera was used and quantification of flow fractions during independent heart beats was measured as previously described^7^. Bitplane Imaris was used to get 3D videos of stacks of adult zebrafish cardiac valve confocal images.

### *In situ* hybridization

*Notch1b* and *klf2a* probes were used to check RNA expression, as previously described^7^, following MTZ treated embryos.

### TUNEL assay

For the combined immunocytochemistry-TUNEL assay, 96hpf embryos and adult fish hearts were fixed overnight in 4%PFA-PBS, embedded in 4% agarose and vibrotome sectioned (130 μm thick sections). Sections were fixed again for 10 min in 4%PFA-PBS, washed with PBS and permeabilized with proteinase K (10 μg/ml) for 10 min. After washing (3 × 5 min) with PBST (1× PBS/0.1% Triton) and blocking with 4%BSA in PBST for 2 hours, sections were incubated with primary antibodies overnight at 4 °C (zn5: Zebrafish International Stock Center, 1:10 for embryos or Eln-2, 1:500 for adult fish). The next day, tissue was washed with PBST (3 × 10 min), rinsed with PBS and stained with TUNEL (*In Situ* Cell Death Detection Kit, Fluorescein, Roche) for 1 h at 37 °C according to the manufacturer’s instructions. After staining, sections were washed with PBST (3 × 5 min) and incubated with secondary antibodies (Alexa anti-mouse 633 for ZN5 or Alexa anti-rabbit 633 for Eln-2). Before imaging, cells were counterstained with 40, 6-diamidino-2-phenylindole (DAPI).

### Quantification and statistical analyses

Statistical details of experiments and exact values of the number of animals used for the analyses can be found at the respective figure legends. Initiation of regeneration was defined as the reoccurrence of *UAS-E1b:NfsB-mCherry* signal in regenerating valves after MTZ treatments.

### Ethical statement

The adult zebrafish regeneration protocol was approved by the BRFAA ethics review board and the Attica Veterinary Department according to the guidelines from Directive 2010/63/EU of the European Parliament on the protection of animals used for scientific purposes (EL25BIO003/5520).

## Supplementary information

Supplementary movie 1

Supplementary movie 2

Supplementary movie 3

Supplementary movie 4

Supplementary movie 5

Supplementary movie 6

Supplementary movie 7

Supplementary movie 8

Supplementary movie 9

Supplementary movie 10

Supplementary figures
