# Supplementary material for: Reactivation of Notch signaling is required for cardiac valve regeneration
Source: Sci Rep. 2019 Nov 5;9:16059. doi: 10.1038/s41598-019-52558-y (PMC6831700; doi:10.1038/s41598-019-52558-y)

Supplementary material

Title: Reactivation of Notch signaling is required for cardiac valve regeneration

Short title: Notch is required for Cardiac Valve regeneration

Authors: Panagiotis Kefalos [1,2], Adamantia Agalou [1], Koichi Kawakami [3], Dimitris Beis [1*].

Affiliations:

[1]Zebrafish Disease Model lab, Center for Experimental Surgery, Clinical and Translational Research, Biomedical Research Foundation, Academy of Athens, GR11527, Greece. Tel:+302106597197; fax:+302106597545.

[2]Department of Biology, University of Patras, GR26504, Greece.

[3]Division of Molecular and Developmental Biology, National Institute of Genetics, and Department of Genetics, SOKENDAI (The Graduate University for Advanced Studies), Mishima 411-8540, Japan. Tel:+81559816740; fax +81559815827.

[*]Correspondence to: dbeis@bioacademy.gr

Original Article

Supplementary material figure legends

Supplementary Figure 1. Expression patterns of the 2 Gal4 transgenic lines used for the valve regeneration studies. A-A'. Expression pattern of a hspGFFDMC73A transgenic embryo 3dpf. (Scale bars: 100 and 200μm respectively) **B.** Confocal stack of the expression pattern of the hspGFFDMC73A Gal4 line. Arrows show atrioventricular canal (AV) and bulbus arteriosus (BA). Scale bar: 20μm. **C.** Confocal z-stack showing partial colocalization (cells shown with arrows) of hspGFFDMC73A with the *Tg(7xTCF-Xla.Siam:nlsmCherry)* Scale bar: 50 microns **D-D'**. Expression pattern of a gSAIzGFFD703A transgenic embryo 3dpf (Scale bars: 100 and 200μm respectively). E. Confocal stack of the expression pattern of gSAIzGFFD703A gal4 series. Arrows show atrioventricular canal (AV) and bulbus arteriosus (BA). Scale bar: 20μm. **F.** Confocal z-stack of the *Tg(7xTCF-Xla.Siam:nlsmCherry)* mesenchymal pattern colocalizing with very few gSAIzGFFD703A cells. Scale bar: 50 microns.

Supplementary Figure 2. Quantification of valve cells' ablation and regeneration in larvae. A. A statistically significant difference in the number of mCherry positive cells shows successful and efficient ablation of cardiac valves in MTZ treated 4dpf hspGFFDMC73A/*UAS-E1b:NfsB-mCherry* embryos ($p<0,001$). B. At 12 dpf, MTZ treated larvae have more mCherry positive cells than 4dpf, indicating proliferation but still significantly less than the untreated ($p<0,01$), showing that the regeneration process is still ongoing at this stage. Two-paired t-test was used for the statistic analysis ($n>5$ for all comparisons)

Supplementary Figure 3. Quantification of the reverse blood flow fraction (% of frames where retrograde blood flow is observed/cardiac beat). MTZ treatment at 4dpf (A) results in an increase of the retrograde blood flow fraction ($p<0,01$, two-paired t-test). By 12dpf, no statistically significant difference can be observed between wild-type and regenerating larvae (n=8 and n=5 animals imaged per sample at each developmental stage respectively) B,C. Snapshot and mean flow pattern for ctrl and MTZ treated embryos respectively at 4dpf E,F. Snapshot and mean flow pattern for ctrl and MTZ treated larvae (8days post treatment) respectively at 12dpf. Retrograde blood flow after "+" prior to next heartbeat was not observed at all 12dpf larvae.

Supplementary Figure 4. Apoptosis detection in MTZ-treated hspGFFDMC73A/ *UAS-E1b:NfsB-mCherry* embryos **using the TUNEL assay.** A-E. Z-stack projection of an untreated hspGFFDMC73A/UAS-*E1b:NfsB*-mCherry embryo stained with TUNEL assay (green) and DmGrasp (blue). F-J. Z-stack projection of an MTZ treated hspGFFDMC73A/UAS-*E1b:NfsB*-mCherry embryo 4dpf. Apoptotic cells are detected at the AV canal and BA areas (arrows). Scale bars: 50 microns.

Supplementary Figure 5. Re-activation of *klf2a* and *notch1b* after chemically induced valve damage. A-A'. *In situ* hybridization with *klf2a* probe of a wild-type 5dpf larva. Scale bar 50 microns. B-B'. Expansion of *klf2a* expression in the heart of an hspGFFDMC73A:*UAS-E1b:NfsB*-mCherry transgenic larva 5dpf, 1 day post treatment with MTZ. Scale bar: 50 microns. C-C'. *In situ* hybridization with *notch1b* probe of a wild-type 5dpf larva. D-D'. Expansion of *notch1b* expression in the heart of an hspGFFDMC73A: *UAS-E1b:NfsB*-mCherry transgenic larva at 5dpf, 1 day post treatment with MTZ. N>30 embryos per treatment.

Supplementary Figure 6. Quantification of valve cells' regeneration with and without Notch inhibition in larvae (related to results of Figure 3). A-B. Quantification of mCherry positive cells at 6dpf (2days post MTZ treatment) in hspGFFDMC73A/*UAS-E1b:NfsB*-mCherry and gSAIzGFFD703A/*UAS-E1b:NfsB*-mCherry larvae shows a significant reduction in the number of regenerating cells with the addition of DAPT following cardiac valve cell ablation. Two-paired t-test was used for the statistic analysis ($n>5$, $p<0,001$ for both GAL4 transgenic lines).

Supplementary Figure 7. Apoptosis detection in MTZ treated hspGFFDMC73A/ *UAS-E1b:NfsB-mCherry* **adult hearts using the TUNEL assay.** A-E. Z-stack projection of an elastin stained adult valve of an untreated hspGFFDMC73A/UAS-*E1b:NfsB*-mCherry.

Numbers (1-4) indicate the 4 leaflets of the adult valve. F-J Z-stack projection of an elastin stained adult valve of an MTZ treated hspGFFDMC73A/UAS-*E1b:NfsB*-mCherry fish, immediately following MTZ treatment (12hours, 5mM). Apoptotic cells (arrows) are detected in the leaflets. Apoptosis is also detectable in what seems to be blood cells. Scale bars: 50 microns.

Supplementary Figure 8. Notch signaling is upregulated following adult cardiac valve injury. A-B. Notch signaling is active as documented by the *Tg(Tp1:venus-PEST)* in an adult hspGFFDMC73A/UAS-*E1b:NfsB*-mCherry/*Tp1:venus-PEST* transgenic fish **C-D**. 3days following MTZ treatment to induce valvular damage Notch reporter is significantly upregulated in the AV valve region. E. The quantification of tp1+ cells shows upregulation of Notch signaling, following cardiac valve injury (n=7 hearts, two-paired t-test analysis, $p<0,01$). Scalebars: 50micron.

Supplementary video 1. Intracardiac flow pattern of a wild-type zebrafish embryo at 4dpf used for the quantifications of Figure 1E and Supplementary Figure 3.

Supplementary video 2. Intracardiac flow pattern shows an increase in the retrograde blood flow fraction of an MTZ treated gSAIzGFFD703A/UAS-*E1b:NfsB*-mCherry zebrafish embryo at 4dpf (1 day following MTZ treatment), used for the quantifications of Figure 1I and Supplementary Figure 3.

Supplementary video 3. Intracardiac flow pattern of a wild-type zebrafish 12dpf larva

Supplementary video 4. Intracardiac flow pattern of a 12dpf gSAIzGFFD703A/UAS-E1b*:NfsB- mCherry* larva treated with MTZ at 3dpf.

Supplementary video 5. 3D reconstruction of an adult valve of a double hspGFFDMC73A/UAS-*E1b:NfsB*-mCherry/ *UAS:eGFP1A* transgenic stained with elastin2.

Supplementary video 6. 3D reconstruction of an adult valve of a hspGFFDMC73A/UAS-*E1b:NfsB*-mCherry/ *UAS:eGFP1A* transgenic treated with MTZ and stained with elastin2.

Supplementary video 7. 3D reconstruction of an adult valve of a *Tp1:venusPEST* transgenic stained with elastin2.

Supplementary video 8. 3D reconstruction of an adult valve of *Tp1:venusPEST* transgenic 3days after MTZ treatment in a *hspGFFDMC73A/UAS-E1b:NfsB--mCherry* transgenic showing upregulation of expression, stained with elastin2.

Supplementary video 9. 3D reconstruction of a regenerating adult valve of a *hspGFFDMC73A/UAS-E1b:NfsB-mCherry/UAS:eGFP1A* transgenic, post MTZ ablation and stained with elastin2.

Supplementary video 10. 3D reconstruction of an adult valve of a *hspGFFDMC73A/UAS-E1b:NfsB-mCherry/UAS:eGFP1A* transgenic treated with DAPT following up the MTZ ablation, and stained with elastin2.

Supplementary Figure 1. Expression patterns of the 2 Gal4 transgenic lines used for the valve regeneration studies.

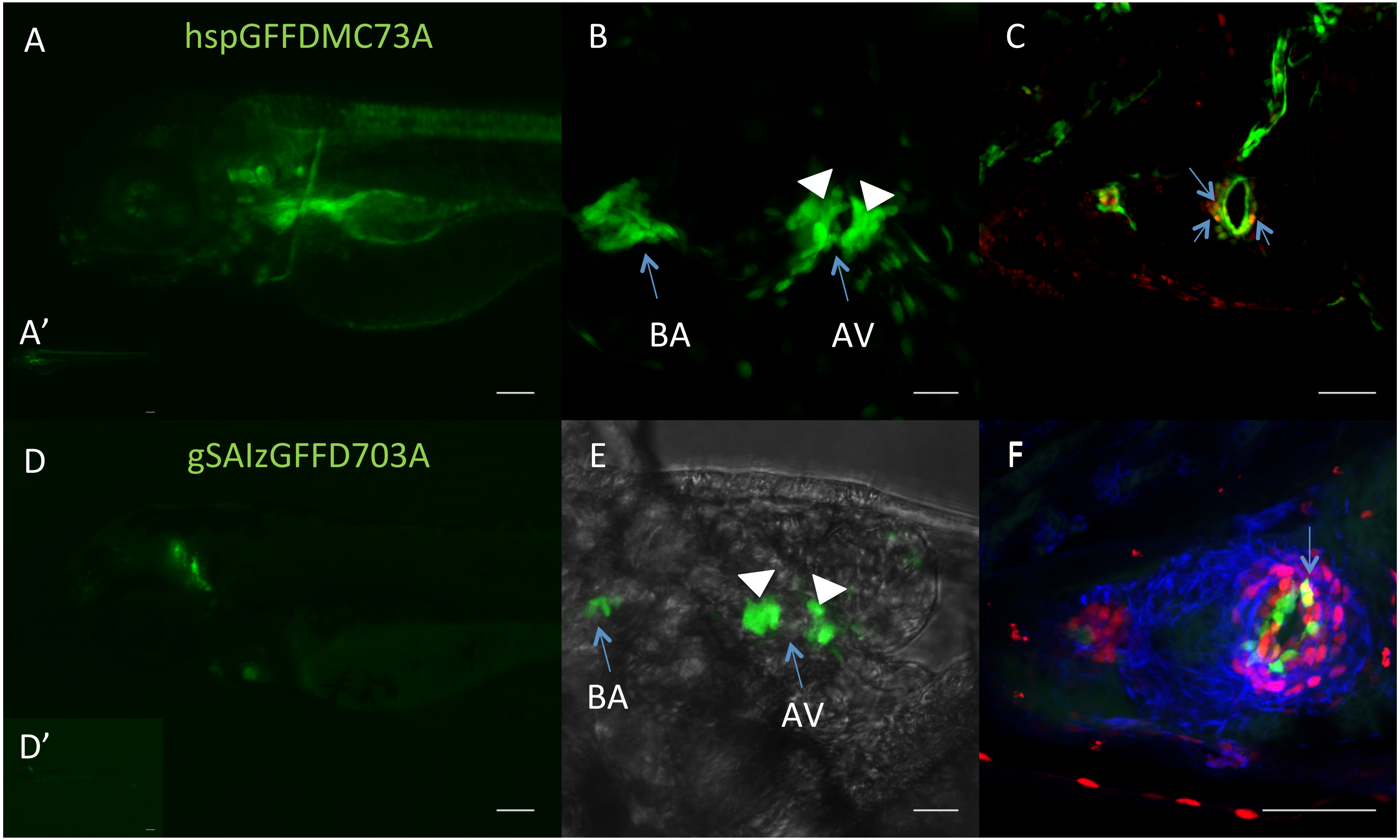

Supplementary Figure 2. Quantification of valve cells' ablation and regeneration in larvae.

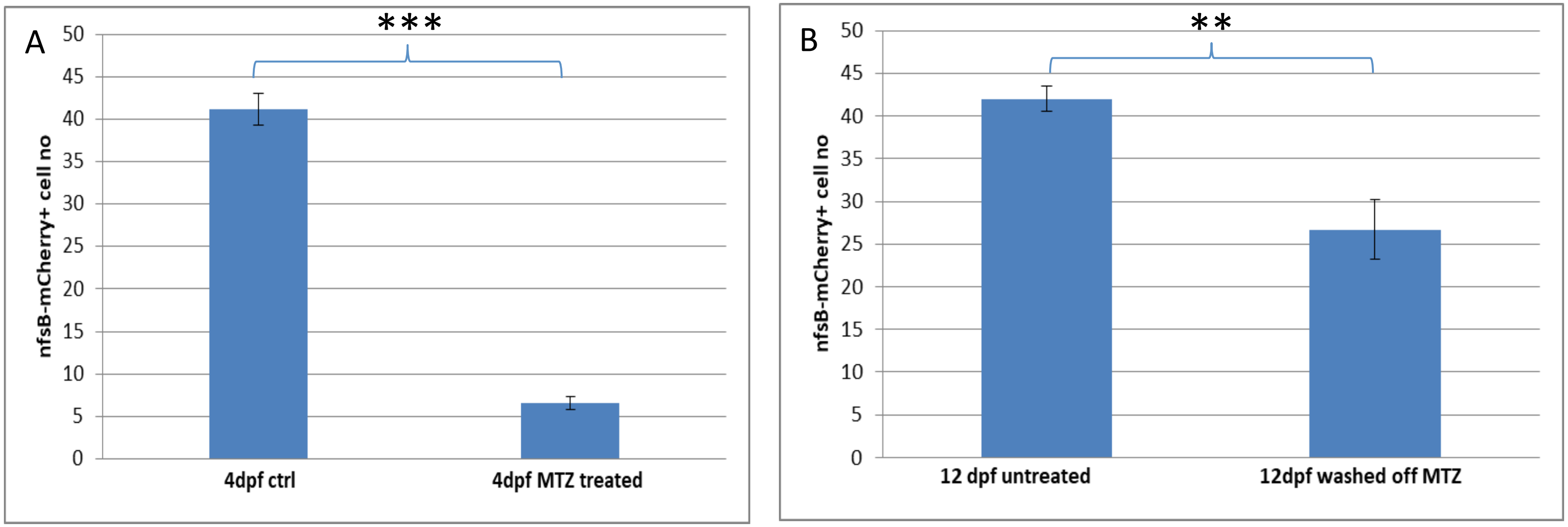

Supplementary Figure 3. Quantification of the reverse blood flow fraction (% of frames where retrograde blood flow is observed/cardiac beat).

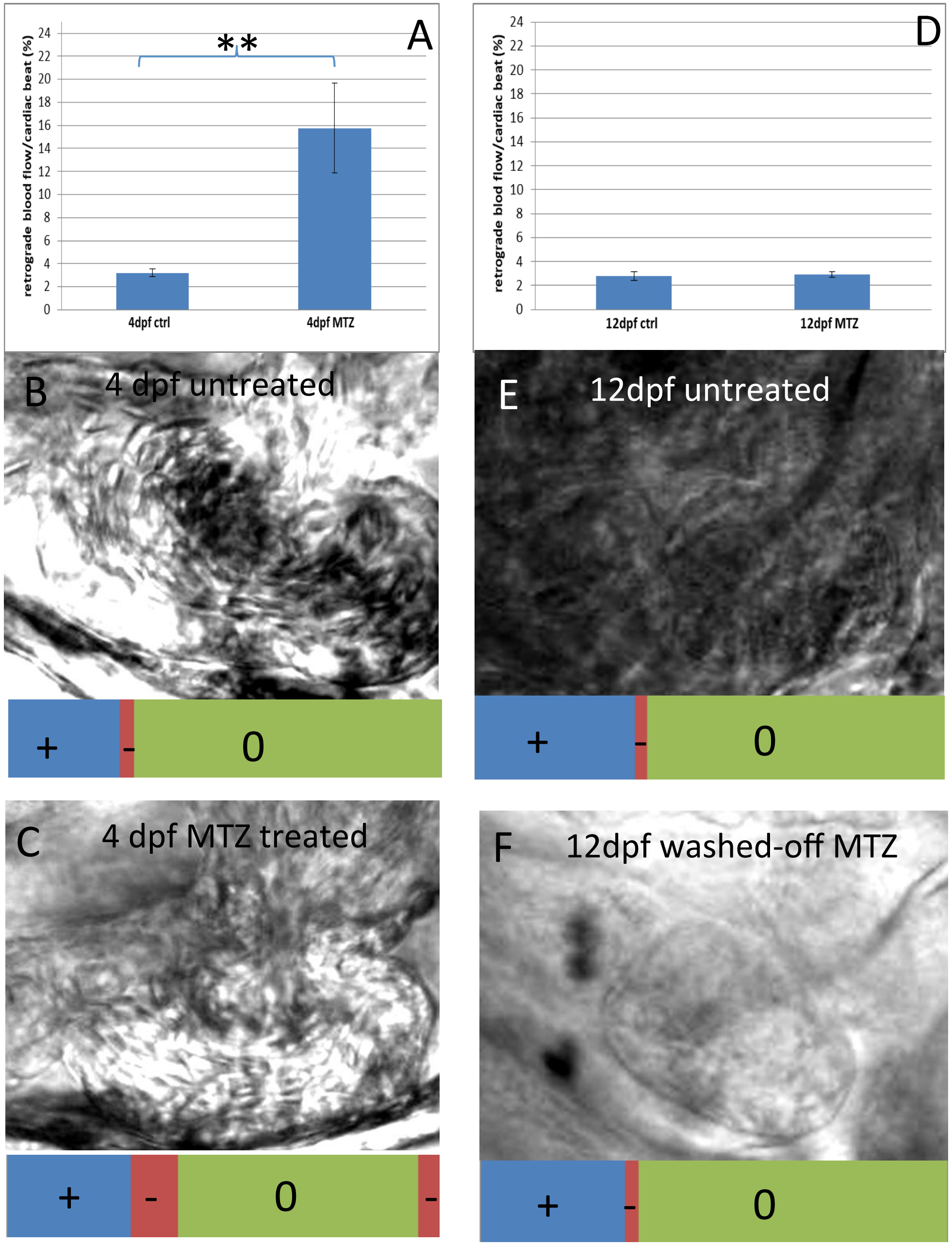

Supplementary Figure 4. Apoptosis detection in MTZ-treated hspGFFDMC73A/ *UAS-E1b:NfsB-mCherry* embryos using the TUNEL assay.

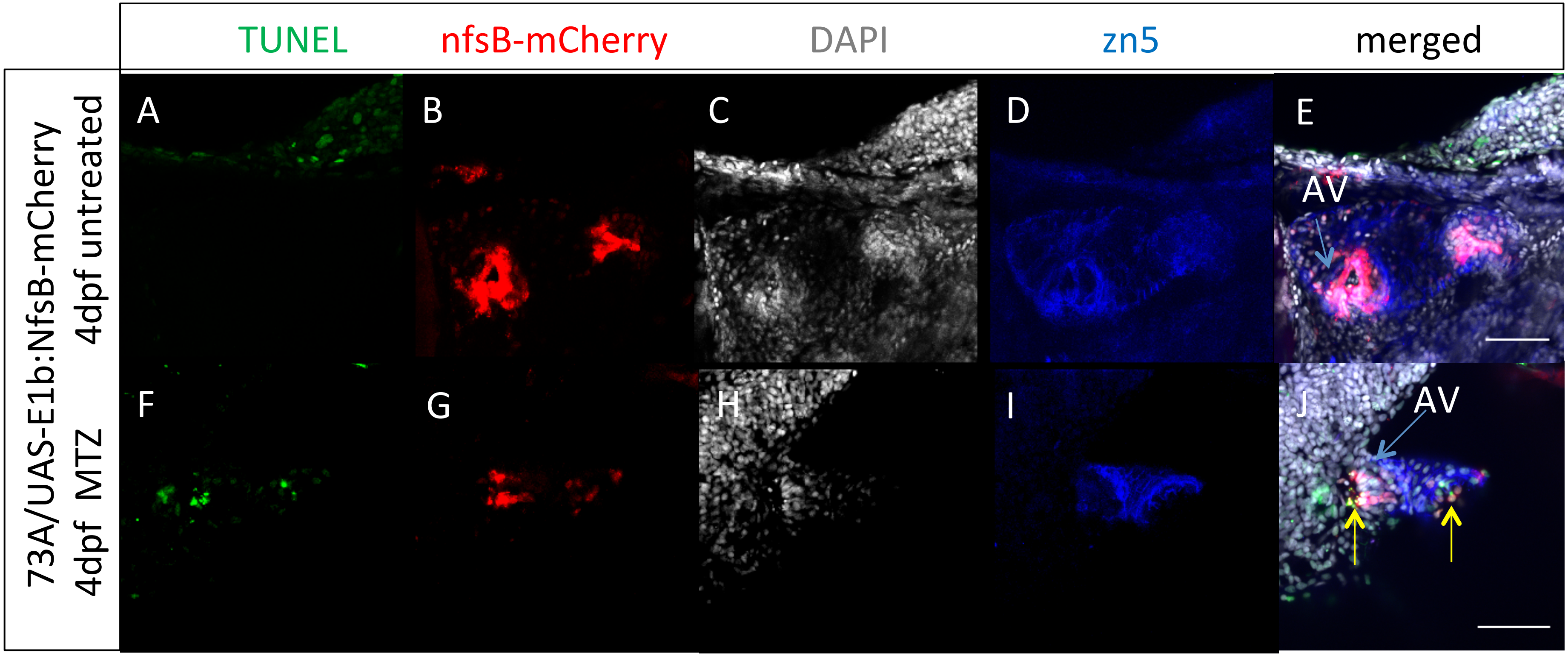

Supplementary Figure 5. Re-activation of *klf2a* and *notch1b* after chemically induced valve damage.

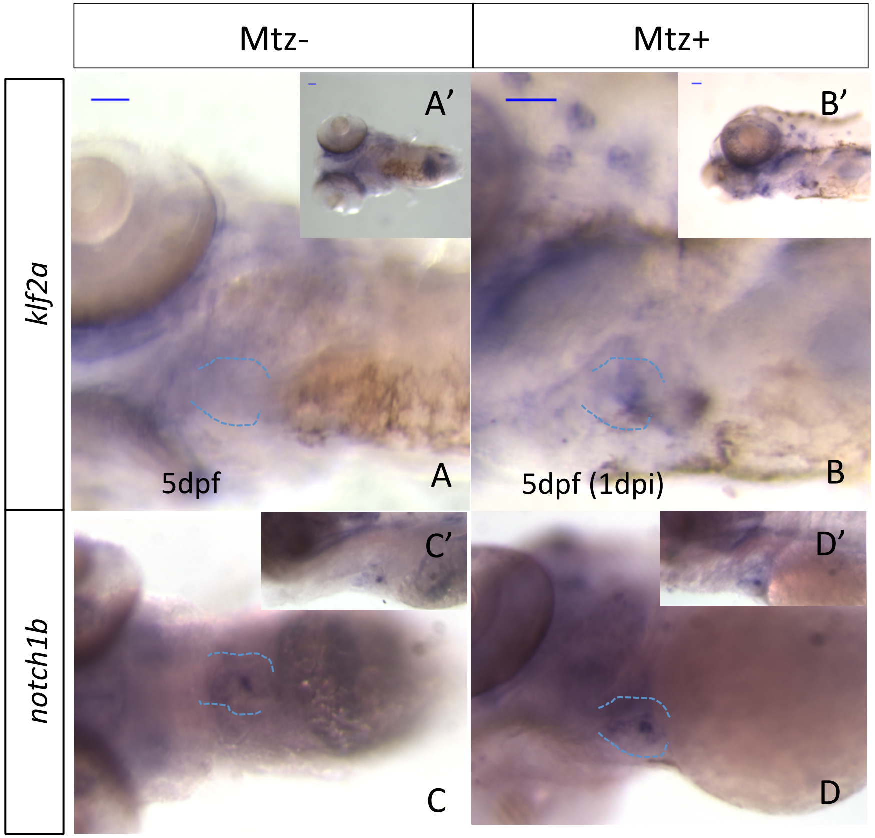

Supplementary Figure 6. Quantification of valve cells' regeneration with and without Notch inhibition in larvae (related to results of Figure 3).

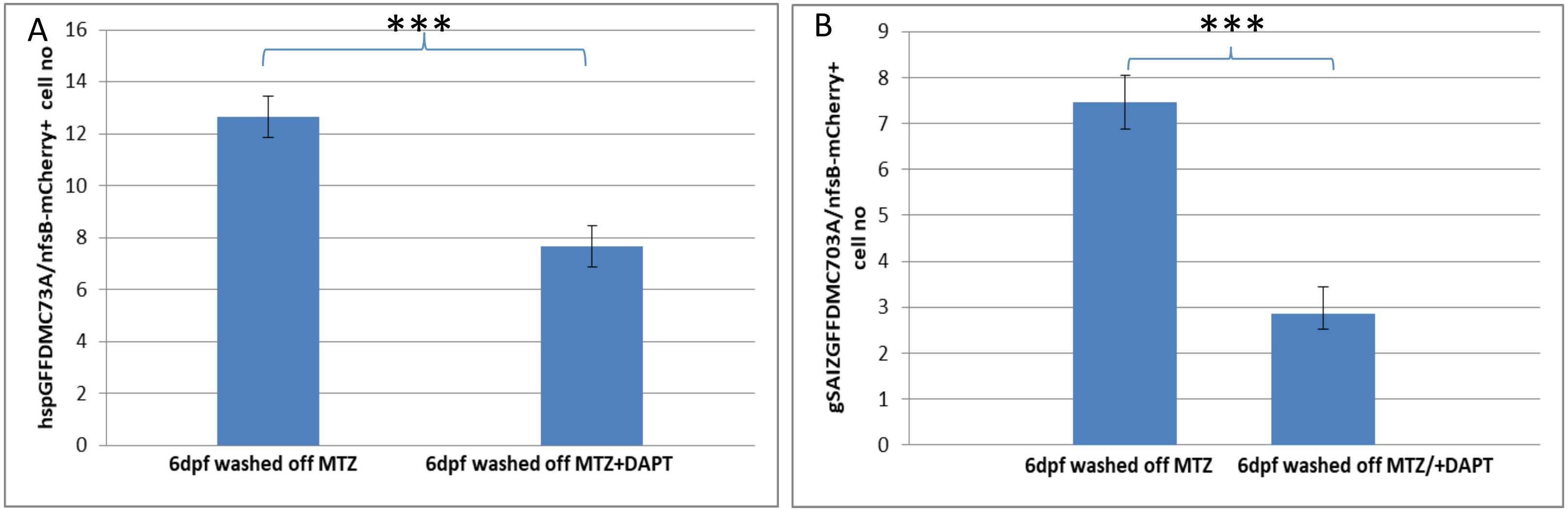

Supplementary Figure 7. Apoptosis detection in MTZ treated hspGFFDMC73A/ *UAS-E1b:NfsB-mCherry* adult hearts using the TUNEL assay.

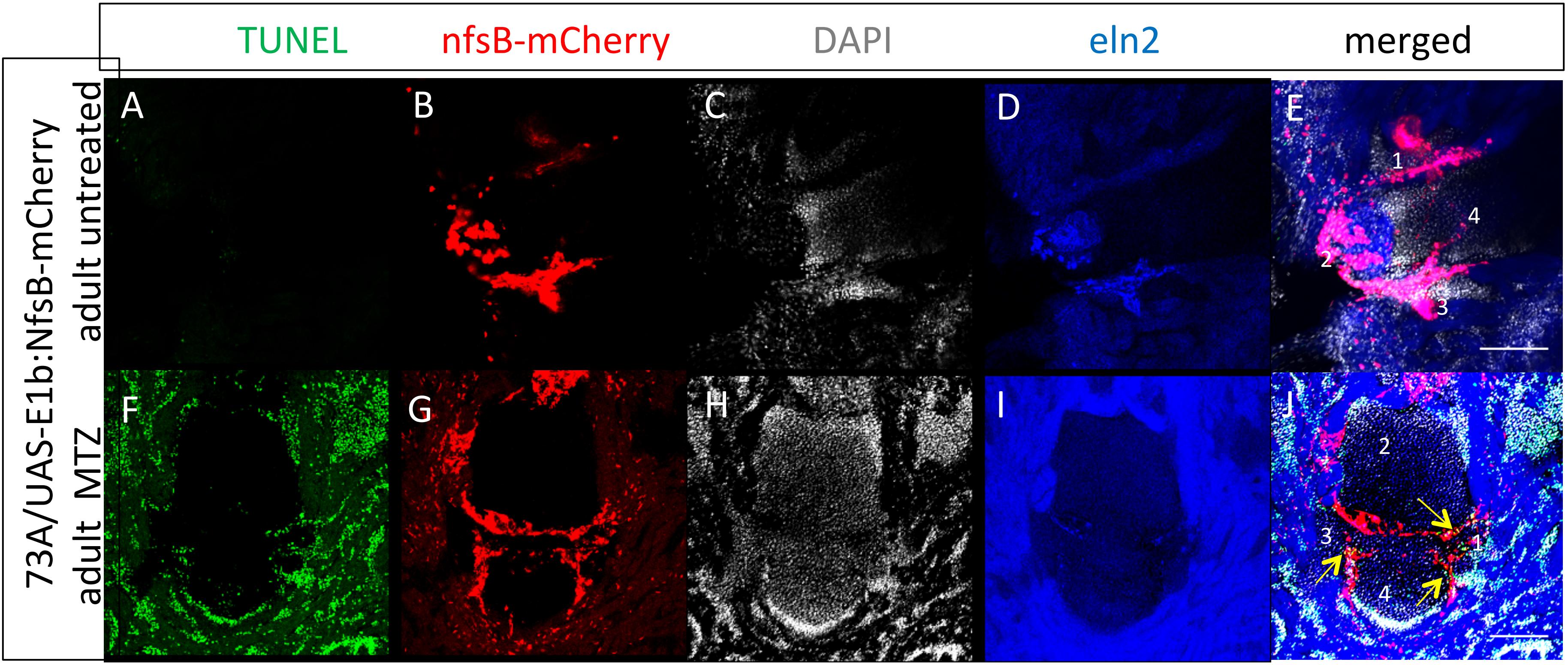

Supplementary Figure 8. Notch signaling is upregulated following adult cardiac valve injury.

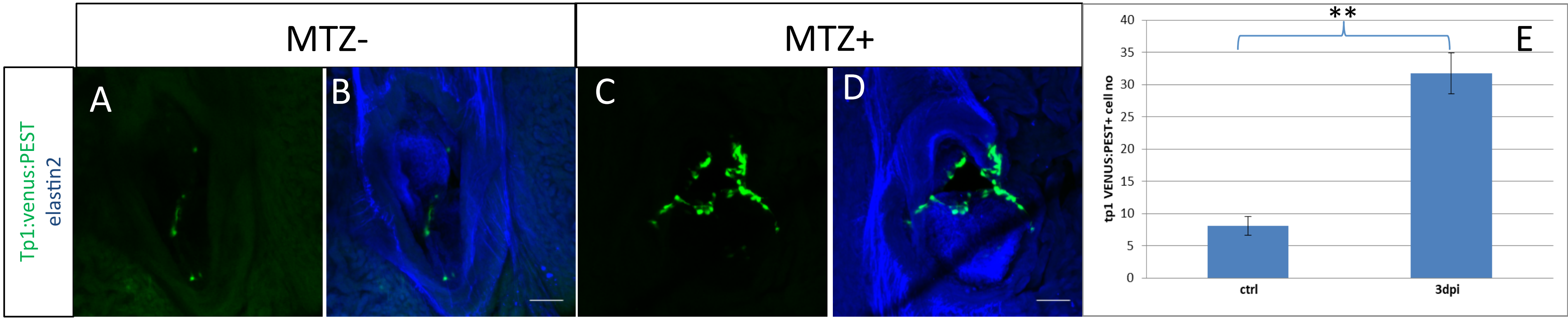

Supplement: Supplementary file 11 — Supplementary figures [file 41598_2019_52558_MOESM11_ESM.pdf]
